# HIV disease progression compared by linkage status in Rwanda and Zambia

**DOI:** 10.1186/1742-4690-9-S2-P161

**Published:** 2012-09-13

**Authors:** W Kilembe, C Dinh, S Lakhi, E Karita, R Bayingana, M Price, S Allen, E Hunter

**Affiliations:** 1 Rwanda Zambia HIV Research Group, Lusaka, Zambia; 2 International AIDS Vaccine Initiative, San Fransisco, CA, USA; 3 Emory University School of Medicine, Atlanta, GA, USA; 4 Emory Vaccine Center and Yerkes Primate Center, Atlanta, GA, USA

## Background

Recently HIV infected individuals are followed up at Rwanda-Zambia HIV Research Group (RZHRG) sites to establish markers of disease progression. Here we compare disease progression between seroconverters infected by their spouses (linked transmission) to those infected by non-spousal partners (unlinked transmission) where a greater fraction of individuals are infected by multiple virus variants.

## Methods

Seroconverters (SC) were identified from HIV discordant couples enrolled from the Couples’ VCT program into a prospective cohort study. Linkage of transmission was established by comparing viral DNA sequences in the SC to that of the suspected index partner. SCs were followed up to six years to collect clinical and laboratory data. Data analysis using STATA was done on CD4 and viral load trajectories and a comparison of disease progression with respect to time to endpoints of CD4 <350 cells/uL and initiation of ARVs. Log rank test of equality for survival function was performed for each endpoint and p-values calculated.

## Results

From February 2006 to December 2011, 88 unlinked and 225 linked transmissions were identified. Across all RZHRG sites, SC males represented 56% of the unlinked and 55% of the linked group. Overall mean age at seroconversion was 33 years and 69% of HIV transmissions were acute infections (identified within 90 days of infections). Mean CD4 count and viral load at specified time points during a six year follow up period of the two groups were similar. Disease progression in the two groups (CD4 <350cell/uL or initiation of ARVs) showed no significant difference (p=0.21 and p=0.26) respectively.

## Conclusion

Despite the fact that previous studies have shown that there is faster disease progression in individuals who were infected by multiple genetic variants from their partner, we show here that linkage status does not predict HIV disease progression in HIV seroconverting couples.

